# Supplementary material for: Ascle: A Python Natural Language Processing Toolkit for Medical Text Generation
Source: ArXiv. 2023 Dec 9:arXiv:2311.16588v2. Preprint. [Version 2] (PMC12478431)
Supplement: Supplement 1 [file NIHPP2311.16588v2-supplement-1.pdf]

Supplementary Appendix

Supplementary Appendix A: Evaluation Guidance for Human Validation

Readability
The quality of the answer text, ignoring the input.

1 (bad): The text is highly difficult to read, full of grammatical errors, and lacks coherence and clarity.

2: The text is somewhat difficult to read, and there are occasional grammatical errors. The coherence and clarity could be improved.<br><br>3: The text is moderately easy to read, but there are noticeable grammatical errors and some parts lack coherence and clarity.<br><br>4: The text is fairly easy to read, with only a few minor grammatical errors. Overall coherence and clarity are good, but there is room for improvement.<br><br>5 (good): The text is easy to read, well-structured, and flows naturally.
**Relevancy**<br>The pertinence of the answer to the posed question.<br><br>1 (bad): The answer is entirely off-topic and does not address the question at all.<br><br>2: The answer somewhat addresses the question but contains a significant amount of irrelevant information.<br><br>3: The answer is moderately relevant to the question but could be more focused.<br><br>4: The answer is mostly relevant with only minor deviations from the topic.<br><br>5 (good): The answer directly addresses the question and stays on topic throughout.
**Accuracy**<br>The correctness and truthfulness of the information provided in the answer.<br><br>1 (bad): The answer contains entirely incorrect or misleading information.<br><br>2: The answer contains several inaccuracies or misleading statements.<br><br>3: The answer is somewhat accurate but has noticeable errors.<br><br>4: The answer is mostly accurate with only minor errors.<br><br>5 (good): The answer is entirely accurate and trustworthy.
**Completeness**<br>The extent to which the answer covers all aspects of the question **(compared with the ground truth)**.<br><br>1 (bad): The answer barely touches on the topic and leaves out most of the necessary information.<br><br>2: The answer covers some aspects of the question but misses several key points.<br><br>3: The answer provides a moderate amount of information but could be more comprehensive.<br><br>4: The answer is fairly comprehensive but misses a few minor details.<br><br>5 (good): The answer thoroughly addresses all aspects of the question and leaves no stone unturned.

Supplementary Appendix B: Machine Translation Evaluation on Other Five Language Pairs

We also fine-tuned mT5 on other five language pairs: "en-cs", "en-de", "en-hu", "en-pl", "en-sv", and evaluated the performance using the BLEU score.

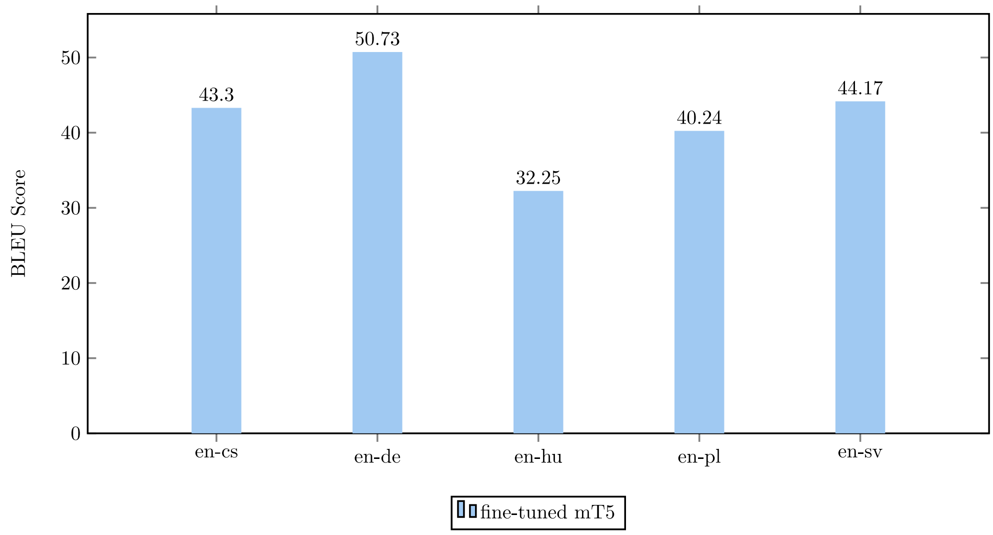

Supplementary Appendix C: Case Study

Based on the physicians' ratings, we selected two typical cases: In the first case, the generated answer is entirely consistent with the ground truth, achieving a score of 5 in readability, relevancy, accuracy, and completeness. In the second case, although the generated answer scored 5 in readability and scored 4 in relevancy, it is incorrect and completely contrary to the ground truth, receiving the lowest scores in accuracy and completeness.

**Question:**<br>What should I do if I forget a dose of Amphetamine ?
**Ground Truth:**<br>Take the missed dose as soon as you remember it. However, if it is almost time for the next dose, skip the missed dose and continue your regular dosing schedule. Do not take a double dose to make up for a missed one.)
**Generated Answer by Baize-healthcare**<br>**(Readability: 5, Relevancy: 5, Accuracy: 5, Completeness: 5)**<br><br>If you forget a dose of Amphetamine, you should take it as soon as you remember. However, if it is almost time for your next dose, skip the missed dose and continue with your regular dosing schedule. Do not take a double dose to make up for the missed one.

**Question:**<br>Is ring chromosome 20 syndrome inherited ? (Also called: r(20) syndrome; ring 20 syndrome; ring

chromosome 20; ring chromosome 20 epilepsy syndrome)
**Ground Truth:**<br>**Ring chromosome 20 syndrome is almost never inherited.** A ring chromosome typically occurs as a random event during the formation of reproductive cells (eggs or sperm) or in early embryonic development. Often, the ring chromosome is present in only some of a person's cells. This situation is known as mosaicism. Most affected individuals have no history of the disorder in their families. However, at least one family has been reported in which a ring chromosome 20 was passed from a mother to her children.)
**Generated Answer by Baize-healthcare**<br>**(Readability: 5, Relevancy: 4, Accuracy: 1, Completeness: 1)**<br><br>**Yes, ring chromosome 20 syndrome is inherited in an autosomal recessive manner,** which means that a person must inherit two copies of the mutated gene (one from each parent) to develop the condition.

Supplementary Appendix D: 12 Basic NLP Functions in Ascle

Functions	Libraries
Abbreviation Extraction	scispaCy
Sentence Tokenization	MedspaCy, scispaCy, Stanza Biomed
Word Tokenization	MedspaCy, scispaCy, Stanza Biomed
Negation Detection	scispaCy
Hyponym Detection	scispaCy
UMLS Concept Extraction	scispaCy
Named Entity Recognition	MedspaCy, scispaCy, Stanza Biomed
Document Clustering	GloVe, Transformers
POS Tagging	MedspaCy, scispaCy, Stanza Biomed
Entity Linking	MedspaCy
Text Summarization	summa
Multi-choice QA	Transformers

Supplementary Appendix E: Evaluation of Basic NLP Functions

POS Tagging

We conducted evaluations on 2019 CRAFT Shared Task (CRAFT-SA) and GENIA corpus. The testing case numbers are 9069 and 2036 respectively. We chose to test on various scispaCy models, a pretrained open-source model (flair/pos-english), and four Stanza Biomed models, with the results shown in the below figure. We concluded that scispaCy models are better on GENIA significantly,

but on CRAFT, they all have a comparable performance.

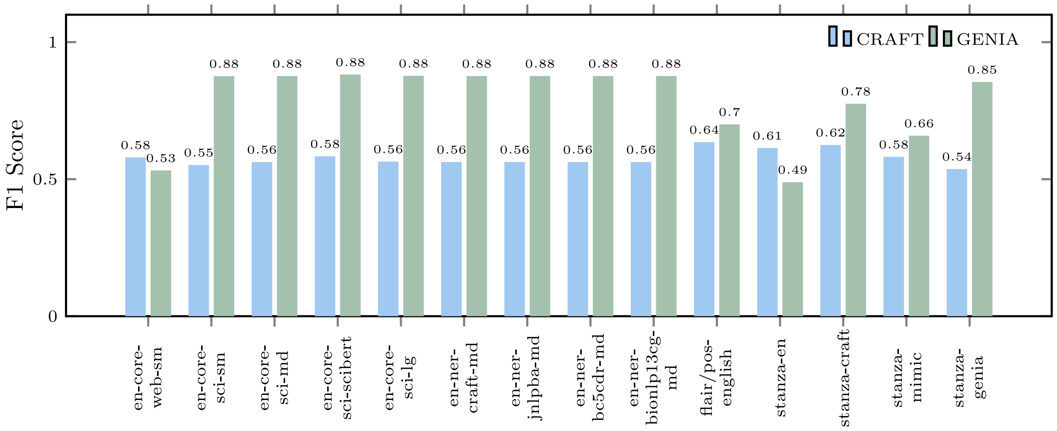

Named Entity Recognition

We chose NCBI disease corpus and BC5CDR (which contains both disease and chemical entity types) datasets for evaluation. The test sets for these two datasets consist of 941 and 4,797 instances, respectively. We compared seven different models from scispaCy and Stanza Biomed, as shown in below table. Stanza models exhibit superior performance, but it is noteworthy that these models were specifically pre-trained for these datasets, whereas in the scispaCy, only "scispaCy-bc5cdr-md" was specifically pre-trained on the BC5CDR dataset.

	NCBI-disease			BC5CDR-disease			BC5CDR-chem		
	R	P	F1	R	P	F1	R	P	F1
scispaCy-sci-sm	61.88	8.07	14.28	74.55	8.76	15.68	73.24	10.48	18.33
scispaCy-sci-md	62.50	7.98	14.16	76.60	8.89	15.94	77.51	10.96	19.20
scispaCy-bc5cdr-md	51.56	52.38	51.97	76.24	36.39	49.27	84.70	49.21	62.25
Stanza-default	**83.44**	82.83	**83.13**	76.51	78.10	77.30	80.58	86.01	83.20
Stanza-mimic	76.98	**84.36**	80.50	75.95	80.02	77.93	76.06	86.74	81.05
Stanza-craft	79.69	84.16	81.86	**81.04**	79.95	80.49	**85.89**	88.33	**87.09**
Stanza-genia	76.56	83.52	79.89	80.42	**81.29**	**80.85**	82.95	**88.49**	85.63
